# The DUSP26 phosphatase activator adenylate kinase 2 regulates FADD phosphorylation and cell growth

**DOI:** 10.1038/ncomms4351

**Published:** 2014-02-19

**Authors:** Hyunjoo Kim, Ho-June Lee, Yumin Oh, Seon-Guk Choi, Se-Hoon Hong, Hyo-Jin Kim, Song-Yi Lee, Ji-Woo Choi, Deog Su Hwang, Key-Sun Kim, Hyo-Joon Kim, Jianke Zhang, Hyun-Jo Youn, Dong-Young Noh, Yong-Keun Jung

**Affiliations:** 1 School of Biological Science/Bio-Max Institute, Seoul National University, Gwanak-gu, Seoul 151-747, Korea; 2 Department of Discovery Oncology, Genentech, Inc. 1 DNA Way, South San Francisco, California 94080, USA; 3 Center for Neuroscience, Korea Institute of Science and Technology, Seoul 136-791, Korea; 4 Department of Biochemistry, Hanyang University, Ansan, Kyeonggi-do 425-791, Korea; 5 Department of Microbiology and Immunology, Thomas Jefferson University, Philadelphia, Pennsylvania 19107, USA; 6 Department of Surgery, Chonbuk National University Medical School, Jeonju 561-180, Korea; 7 Department of Surgery, Seoul National University College of Medicine, Seoul 110-744, Korea; 8 These authors contributed equally to this work

## Abstract

Adenylate kinase 2 (AK2), which balances adenine nucleotide pool, is a multi-functional protein. Here we show that AK2 negatively regulates tumour cell growth. AK2 forms a complex with dual-specificity phosphatase 26 (DUSP26) phosphatase and stimulates DUSP26 activity independently of its AK activity. AK2/DUSP26 phosphatase protein complex dephosphorylates fas-associated protein with death domain (FADD) and regulates cell growth. AK2 deficiency enhances cell proliferation and induces tumour formation in a xenograft assay. This anti-growth function of AK2 is associated with its DUSP26-stimulating activity. Downregulation of AK2 is frequently found in tumour cells and human cancer tissues showing high levels of phospho-FADD_Ser194_. Moreover, reconstitution of AK2 in AK2-deficient tumour cells retards both cell proliferation and tumourigenesis. Consistent with this, *AK2*^+/−^ mouse embryo fibroblasts exhibit enhanced cell proliferation with a significant alteration in phospho-FADD_Ser191_. These results suggest that AK2 is an associated activator of DUSP26 and suppresses cell proliferation by FADD dephosphorylation, postulating AK2 as a negative regulator of tumour growth.

## Abstract (web summary)

Adenylate kinase 2 can bind to FADD. In this study, Kim *et al.* show that adenylate kinase 2 is a tumour suppressor and interacts with the phosphatase DUSP6, and this in turn regulates the phosphorylation of FADD.

## Graphical abstract

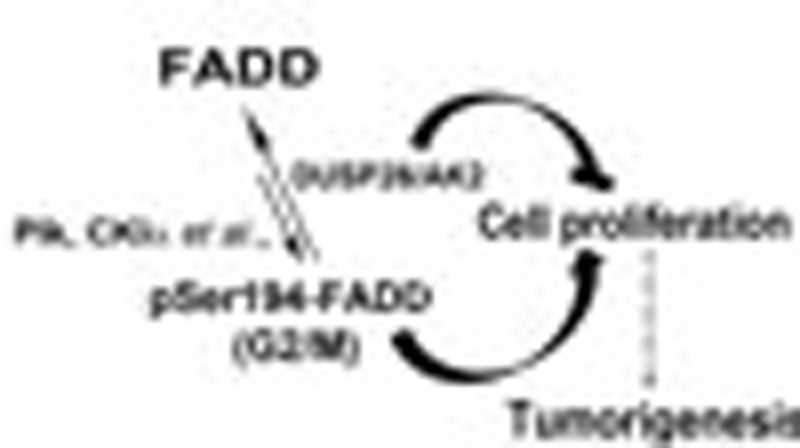

Adenylate kinase (AK) is a ubiquitous enzyme that catalyses the nucleotide phosphoryl exchange reaction^1^. Various isozymes of AK have so far been characterized and are located in different tissues and subcellular region^2^,^3^. Among them, AK2 (AK2/ATP–AMP transphosphorylase) is a metabolic enzyme often linked to mitochondrial metabolic rate and has been proposed to have a role in mitochondria–nucleus energetic signal communication^2^,^4^. Recent studies have highlighted new exciting developments regarding multi-faceted AK2^5^. AK2 is released into the cytosol to form an apoptotic complex upon initiation of cell death^5^,^6^. Moreover, human AK2 deficiency causes a hematopoietic defect associated with sensorineural deafness^7^,^8^ and Keloids disease associated with fibroproliferative tumours developing as a result of deregulated wound healing. AK2 was also proposed to associate with mitotic spindle apparently to power cell division cycle^3^, and its dysregulation was observed in human cancers and amyotrophic lateral sclerosis (ALS) diseases^9^,^10^,^11^,^12^. Thus, AK2 may have important extra-mitochondrial functions, especially in tumourigenesis.

Dual-specificity phosphatase 26 (DUSP26) is an atypical DUSP phosphatase that is a heterogeneous group of protein phosphatases, and can dephosphorylate both phosphotyrosine and phosphoserine/phosphothreonine residues within one substrate^13^. DUSP26 functions as a p38 phosphatase and an extracellular signal-regulated kinase (ERK) phosphatase, as well as a positive regulator of c-Jun NH(2)-terminal kinase (JNK)^14^,^15^,^16^,^17^. In addition, DUSP26-interacting partners, including KIF3 and p53, have been identified^18^,^19^. It was also suggested that DUSP26 regulates cell proliferation in neuronal cells^20^,^21^ and exhibits loss of heterozygosity in several malignancies, including breast, prostate and ovarian cancers^22^,^23^,^24^. However, molecular detail by which DUSP26 activity is modulated and how DUSP26 regulates cell growth are unknown.

Besides well-known roles in the extrinsic and intrinsic apoptosis^5^,^25^,^26^, increasing evidence showed that FADD has essential functions in embryogenesis, nuclear factor-KappaB (NF-κB) activation, cell cycle progression, cell proliferation and tumourigenesis^27^,^28^,^29^,^30^,^31^. The locus of *FADD* on chromosome 11q13.3 is a hot spot for chromosomal amplification in a number of human cancers^32^. Especially, *FADD* knockout T cells and a dominant-interfering mutant of FADD show impaired cell proliferation. In particular, many of these non-apoptotic activities are determined by the phosphorylated (p)-FADD at Ser194 (mouse: Ser191 and human: Ser194) in a region distinct from the pro-apoptotic function. FADD undergoes cell cycle-dependent phosphorylation at Ser194, through which it may regulate cell cycle progression^32^,^33^. Mice bearing an Asp mutation at Ser191 exhibit problems with immune system development indicative of proliferative defects^34^. Furthermore, high levels of p-FADD have been detected in several cancer cell types^34^,^35^,^36^,^37^ and reportedly associated with tumourigenesis^32^,^38^,^39^. Although several kinases responsible for FADD phosphorylation have been intensely investigated, such as Fas/FADD-interacting serine/threonine kinase (FIST/HIPK3), a 70-kDa cell cycle-regulated kinase, protein kinase C-ζ, polo-like kinase 1 and CKI-α^33^,^39^,^40^,^41^,^42^, the molecular events involved in reversing FADD phosphorylation remain unknown, highlighting the need to understand the mechanism underlying the function of the multi-faceted FADD.

In the present study, we show that AK2 forms a protein complex with DUSP26 and stimulates the phosphatase activity of DUSP26 resulting in the dephosphorylation of p-FADD and the regulation of tumour cell growth. Loss of AK2 expression is associated with rapid cell proliferation and often found in breast cancers, providing a molecular basis for the role of AK2/DUSP26 complex as a potent regulator of tumour growth.

## Results

### Nuclear AK2 negatively regulates FADD phosphorylation

**Figure 1:**
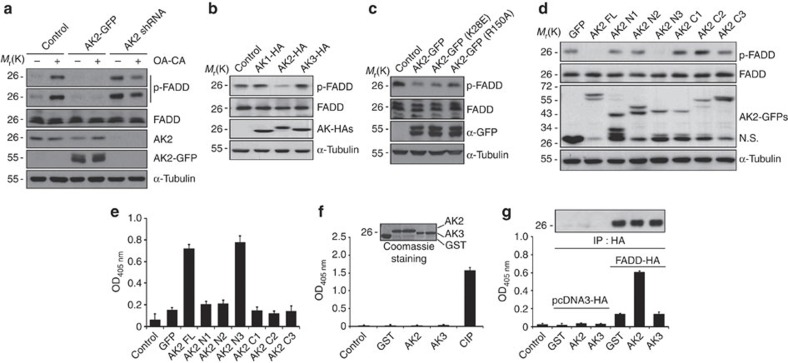
AK2 regulates FADD phosphorylation. (**a**) AK2-dependent regulation of FADD phosphorylation. HeLa cells were transfected with pEGFP (Control), pAK2-GFP or pAK2-shRNA for 24 h and then left untreated or treated with okadaic acid (OA, 0.5 μM) and calyculin A (CA, 50 nM) for 2 h. Cell extracts were analysed by western blotting with the indicated antibodies. (**b**) Ectopic expression of AK2, but not AK1 or AK3, dephosphorylates FADD. HeLa cells were transfected with pAK1-HA, pAK2-HA or pAK3-HA for 24 h, after which cell extracts were subjected to western blotting with anti-p-FADD, anti-FADD or anti-HA antibodies. (**c**) The ability of AK2 to dephosphorylate FADD is independent of its enzyme activity. HEK293T cells were transfected with pcDNA (Control), pAK2-GFP, pAK2 K28E-GFP or pAK2 R150A-GFP for 24 h, and cell extracts were then prepared and subjected to western blotting. (**d**) The N-terminal and middle regions of AK2, comprised of the NMPbind and LID domains (AK2 N3), mediate FADD dephosphorylation. HeLa cells were transfected with pAK2 or AK2 deletion mutants for 24 h, and cell extracts were subjected to western blotting. NS indicates non-specific signal. (**e**) The AK2 N3 mutant exhibits phosphatase activity. HEK293T cells were co-transfected with p-FADD-HA and either pAK2 or AK2 deletion mutants for 24 h. After fractionation of cell extracts, nuclear fractions were subjected to immunoprecipitation (IP) using anti-HA antibody, after which the immunoprecipitates were analysed for the phosphatase activity using pNPP. (**f**) Purified AK2 protein does not exhibit a phosphatase activity. Bacterially purified GST, His-AK2, His-AK3 and calf intestinal phosphatase (CIP) proteins were incubated with pNPP for 15 min and the OD_405nm_ of the reaction supernatants was measured with spectrophotometer. Purified proteins used in these assays were stained with Coomassie-blue (insert). (**g**) Purified AK2 protein enhances the phosphatase activity of FADD-containing protein complexes. HEK293T cells were transfected with pcDNA-HA or p-FADD-HA for 24 h, after which cell extracts were subjected to IP assay using anti-HA antibody. Half of the immunocomplex was analysed by western blotting with anti-HA antibody (insert) and the other half was incubated with purified GST, His-AK2 or His-AK3 protein (each 10 μg) for 1 h. Then, the reactions were examined for phosphatase activity using pNPP. Bars represent mean±s.d. (*n*=3).

Based on our earlier report showing that AK2 binds to FADD, we addressed whether AK2 is responsible for the regulation of FADD phosphorylation. Reduction of AK2 expression apparently enhanced the level of p-FADD, whereas ectopic expression of AK2 reduced it (Fig. 1a). As reported, we confirmed the increase of this FADD phosphorylation after treating cells with phosphatase inhibitors, okadaic acid and calyculin A, and no phosphorylation of an FADD S194A mutant in which Ser194 was replaced with an Ala (Supplementary Fig. 1a). In addition, western blot analysis following two-dimensional PAGE confirmed that the appearance of only one p-FADD that migrated more slowly to a more acidic pH than non-phosphorylated FADD was exclusively regulated by AK2 (Supplementary Fig. 1b). Unlike AK2, ectopic expression of cytosolic AK1 or mitochondrial AK3 had no effect on FADD phosphorylation (Fig. 1b). Moreover, a nucleotide kinase-dead mutant, AK2 K28E (ref. 5), reduced FADD phosphorylation as effectively as wild-type AK2 (Fig. 1c). The ability of AK2 to regulate FADD phosphorylation was also observed in Chang liver and Huh-7 tumour cells (Supplementary Fig. 1c). It thus appears that the activity responsible for FADD dephosphorylation is a unique feature of AK2 among AK isotypes and differs from its AK activity.

To better understand a molecular function of AK2, we used a set of AK2 deletion mutants^5^ and determined AK2 domain responsible for the regulation of FADD dephosphorylation (Fig. 1d). All of the AK2 constructs were fused with green fluorescence protein (GFP), enabling us to visualize their subcellular localization^5^. From ectopic expression analysis, we found that the AK2 N3 mutant comprising the N-terminal NMPbind (amino-acid residues 45–74) and middle LID (amino-acid residues 141–178) regions exhibited the phosphatase activity against p-FADD, which was comparable to that of wild-type AK2 (Fig. 1d). As seen with the AK2 N2 and AK2 C2 mutants, however, further deletion of the NMPbind domain (AK2 C2) or LID domain (AK2 N2) from the AK2 N3 abolished the enzymatic activity to regulate FADD phosphorylation (Fig. 1d).

Despite its proposed localization in mitochondria, an amino-acid homology search suggests a high probability of nuclear AK2 localization (34.8%; PSORT II programme). We therefore examined subcellular distribution of AK2 using fractionation analysis. Although the majority of AK2 localized in the mitochondrial fraction, a significant amount of AK2 was also detected in the nuclear fraction of HeLa cells (Supplementary Fig. 2a). When we isolated AK2/FADD complexes from nuclear fraction with immunoprecipitation assay and assessed phosphatase activity of the immunoprecipitates using universal substrate para-nitrophenylphosphate (pNPP), the protein complexes containing AK2 or AK2 N3 mutant exhibited higher phosphatase activity than other AK2 mutants (Fig. 1e). These results suggest that AK2-associated phosphates activity is present in the nuclear fraction. In keeping with this notion, when we enforced AK2 to be located exclusively in the mitochondria by adding additional mitochondria-targeting sequence of cytochrome *c* oxidase subunit VIII, the mitochondria-targeted AK2 failed to reduce the level of p-FADD in cells (Supplementary Fig. 2b). Besides, p-FADD detected in the nucleus was augmented upon short hairpin RNA (shRNA)-mediated repression of nuclear AK2 (Supplementary Fig. 2c).

Next, we determined whether AK2 itself has a phosphatase activity. Human AK2 protein was expressed in and purified from bacteria, after which enzymatic activity of the purified protein was assessed *in vitro*. However, the purified AK2 protein exhibited no phosphatase activity towards pNPP (Fig. 1f) and a synthetic phospho-Ser_194_ peptide (_**191**_GAM_P_**S**PMS_**197**_) derived from the human FADD (Supplementary Fig. 3a,b). On the contrary, when FADD containing protein complexes were isolated from HEK293T cells by immunoprecipitation assay and then incubated with the AK2 protein, phosphatase activity retained in the FADD-containing immunoprecipitates increased 4- to 5-fold (Fig. 1g) and FADD dephosphorylation was enhanced by AK2 protein (Supplementary Fig. 3c). Unlike AK2, addition of AK3 protein had no effect on the phosphatase activity, consistent with the results shown in Fig. 1b. These results suggest that AK2 itself is not a phosphatase but stimulates phosphatase activity within the FADD-containing protein complexes.

### AK2 binds to DUSP26 and promotes its interaction with FADD

**Figure 2:**
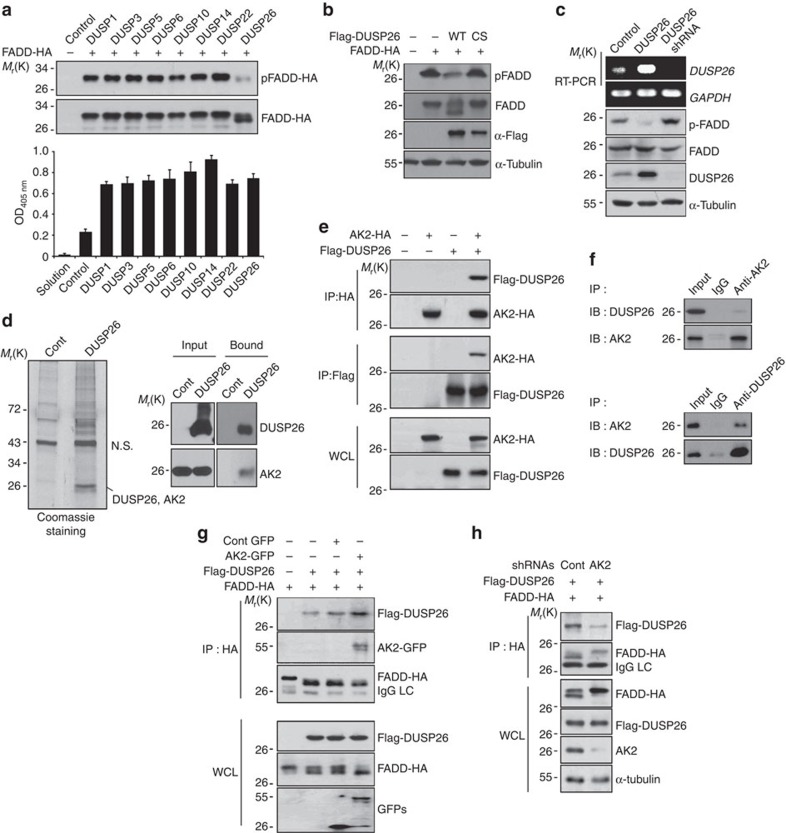
Isolation of DUSP26 as a FADD phosphatase and an AK2-binding partner. (**a**) HEK293T cells were co-transfected with p-FADD-HA and each phosphatase cDNA for 24 h, cell extracts were subjected to western blotting using anti-p-FADD and anti-FADD antibodies and to phosphatase assays with pNPP. Bars represent mean±s.d. (*n*=3). (**b**) HEK293T cells were co-transfected with FADD-HA and pcDNA3, Flag-DUSP26 or Flag-DUSP26 C152S for 24 h, and cell extracts were then analysed by western blotting. (**c**) HeLa cells were transfected with pSuper, pDUSP26 or pDUSP26 shRNA for 36 h, after which cell extracts were subjected to western blotting using anti-p-FADD, anti-FADD and anti-α-tubulin antibodies. Total RNAs were purified and analysed with RT–PCR using DUSP26- or GAPDH-specific synthetic oligonucleotides as primers. (**d**) HEK293T cells were transfected with p3 × Flag or p3 × Flag-DUSP26 for 36 h, and cell extracts were prepared and subjected to the pull-down assay using anti-FLAG agarose-beads. The bound proteins were resolved by SDS–PAGE, stained with Coomassie-blue, and analysed by LC-MS/MS or analysed by western blotting using the indicated antibodies. NS indicates non-specific signal. (**e**) HEK293T cells were co-transfected with pAK2-HA and pFlag-DUSP26 for 36 h and cell extracts were subjected to immunoprecipitation (IP) assay using anti-HA or anti-Flag antibody. Whole-cell lysates and the immunoprecipitates were probed by western blotting with anti-FLAG or anti-HA antibody. (**f**) HeLa cell extracts were subjected to IP analysis using anti-AK2 or anti-DUSP26 antibodies and then the immunoprecipitates were analysed by western blotting with the indicated antibodies. (**g**) AK2 over-expression enhances the binding of DUSP26 to FADD. HEK293T cells were co-transfected with Flag-DUSP26, FADD-HA and either GFP or AK2-GFP for 36 h, after which cells extracts were subjected to IP analysis using anti-HA antibody. Expression levels of Flag-DUSP26, FADD-HA, GFP and AK2-GFP in whole-cell lysates were examined by western blotting with the indicated antibodies. (**h**) HEK293T cells were co-transfected with Flag-DUSP26, FADD-HA and either pSuper or AK2 shRNA for 36 h. Cell lysates were subjected to IP analysis with anti-HA antibody, and expression levels of Flag-DUSP26, FADD-HA and AK2 in whole-cell lysates were examined by western blotting.

Data provided above led us to ask whether AK2 might act as a regulatory protein that could stimulate the phosphatase activity responsible for FADD dephosphorylation. To address this point, we collected 107 cDNAs encoding Ser/Thr phosphatase, including PPM phosphatase, PPP phosphatase, Ser/Thr DUSP and pyrophosphatase, and carried out functional screening assays. Consistent with earlier reports^32^,^40^, FADD frequently showed a migration shift on western blots with the slowly migrating band corresponding to p-FADD (Fig. 2a). Through western blot analysis following co-expression of FADD-HA and each of the 107 Ser/Thr phosphatases, we were able to isolate DUSP26 as a strong candidate phosphatase for FADD. Among DUSPs, ectopic expression of DUSP26 reduced the level of p-FADD in HEK293T cells and increased the level of the fast-migrating FADD (Fig. 2a). Expressions of DUSPs used here were confirmed by their enhanced phosphatase activities in cell extracts (Fig. 2a). In contrast to wild-type DUSP26, expression of phosphatase-dead (C152S) DUSP26 mutant had no effect on the level of p-FADD (Fig. 2b). Conversely, knockdown of DUSP26 expression using shRNA increased the levels of p-FADD (Fig. 2c). Moreover, DUSP26 was also detected in the nuclear fraction of HeLa cells (Supplementary Fig. 4). These results indicate that DUSP26 may be a phosphatase responsible for FADD dephosphorylation in growing cells.

In order to clarify a close connection between AK2 and DUSP26, we attempted other approach to identify the proteins that physically interacted with DUSP26. The pull-down assays using 3 × FLAG-tagged DUSP26 protein revealed that a few proteins with molecular weight 26–28 kDa bound to DUSP26, as visualized by Coomassie-blue staining (Fig. 2d). Liquid chromatography-tandem mass spectrometry (LC-MS/MS) analysis confirmed that the protein bands with 26–28 kDa on the protein gel corresponded to AK2 and DUSP26. Western blot analysis also exhibited the presence of AK2 in the 3 × FLAG-DUSP26-containing protein complexes (Fig. 2d). Consistently, purified AK2 protein bound to ^35^S-methionine-labeled DUSP26 *in vitro* (Supplementary Fig. 5a) and the interaction between over-expressed AK2 and FADD was readily detected in HEK293T cells (Fig. 2e). Further, we found that endogenous AK2 interacted with DUSP26 in HeLa cells and *vice versa* (Fig. 2f), and this interaction was evident especially in the nuclear-enriched fraction of growing cells (Supplementary Fig. 5b). Given that FADD is phosphorylated in G2/M phase of cell cycle^3^, we increased cell population in G2/M phase by treating cells with nocodazole, a microtubule destabilizer, and examined the complex formation. Interestingly, we found that DUSP26 preferentially bound to AK2 and FADD in nocodazole-treated and cell cycle-arrested cells, but not in cells undergoing etoposide-induced cell death (Supplementary Fig. 5c). All of these observations suggest that AK2 forms a novel protein complex with DUSP26 in cells that may depend on cell cycle progression.

To gain insight into the molecular mechanism by which AK2 modulates DUSP26-mediated FADD dephosphorylation, we examined whether AK2 affected the interaction of DUSP26 with its substrate. Immunoprecipitation assays showed that AK2 over-expression enhanced the interaction between DUSP26 and FADD (Fig. 2g), whereas AK2 knockdown markedly reduced it (Fig. 2h). It is also noteworthy that AK2 knockdown resulted in a concomitant accumulation of p-FADD retained in DUSP26 complexes (Fig. 2h). To determine the structural network of AK2/DUSP26 complex and its substrate FADD, we performed domain mapping analysis for their interactions. Detailed binding assays using deletion mutants indicated that the middle region of AK2 containing NMPbind is required for its binding to DUSP26 (Supplementary Fig. S5d) and the N- or C terminus of AK2 interacted with death domain of FADD^5^, whereas DUSP26 bound to death effector domain of FADD (Supplementary Fig. 5e). These observations suggest that AK2 may also participate in and strengthen the protein–protein interaction between AK2/DUSP26 and FADD, allowing FADD to be better recruited into AK2/DUSP26 complex.

### AK2 directly enhances the phosphatase activity of DUSP26

**Figure 3:**
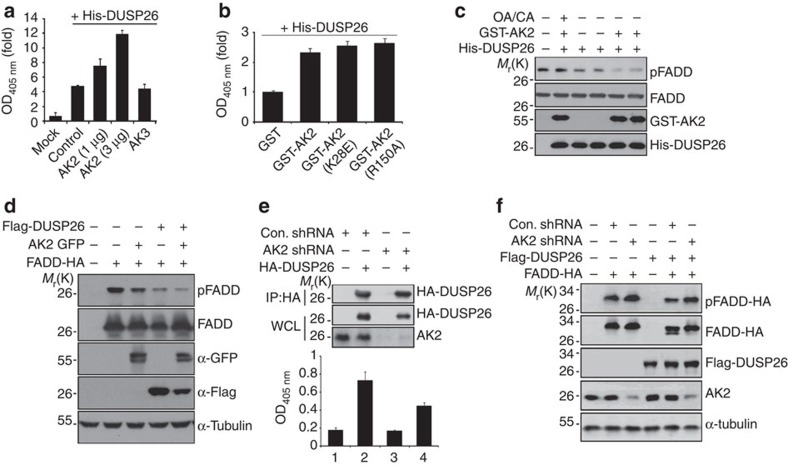
AK2 enhances the phosphatase activity of DUSP26 *in vitro* and in cells. (**a**) Purified AK2 protein enhances the phosphatase activity of DUSP26 *in vitro*. His-DUSP26 protein was purified from bacteria and incubated for 30 min with His-AK2 or His-AK3 protein (each 3 μg). Phosphatase assays were performed with pNPP. Bars represent mean±s.d. (*n*=3). (**b**) AK-dead mutant enhances the phosphatase activity of DUSP26. His-DUSP26 protein was incubated with GST, GST-AK2, GST-AK2 K28E or GST-AK2 R150A protein for 30 min and then subjected to phosphatase assay. Bars represent mean±s.d. (*n*=3). (**c**) AK2 facilitates DUSP26-mediated dephosphorylation of p-FADD. His-FADD protein was phosphorylated by Plk kinase *in vitro* and then incubated at 37 °C for 1 h with GST-AK2, His-DUSP26 alone or both GST-AK2 and His-DUSP26 (each 5 μg) in the presence or absence of okadaic acid (OA) and calyculin A (CA). The reaction mixtures were then subjected to western blotting with anti-AK2, anti-FADD, anti-p-FADD and anti-DUSP26 antibodies. (**d**) Ectopic expression of AK2 enhances DUSP26-catalysed FADD dephosphorylation in cells. HEK293T cells were co-transfected with combinations of the indicated vectors for 12 h, after which cell extracts were subjected to western blotting. (**e**) Reduction of AK2 expression decreases the phosphatase activity of DUSP26-containing protein complexes. HeLa/Cont shRNA and HeLa/AK2 shRNA cells were transfected with pHA-DUSP26 for 36 h, after which cell extracts were subjected to immunoprecipitation (IP) assay with anti-HA antibody. Halves of the immunoprecipitates were then probed by western blotting with anti-AK2 and anti-HA antibodies and the other halves were assayed for phosphatase activity. Whole-cell lysates (WCL) were included for the comparison of AK2 and HA-DUSP26 expression. Bars represent mean±s.d. (*n=*3). (**f**) Downregulation of AK2 expression reduces DUSP26-catalysed FADD dephosphorylation. HEK293T cells were co-transfected with p-FADD-HA, pFlag-DUSP26 and either pSuper (Control) or pAK2-shRNA for 24 h, after which cell extracts were subjected to western blotting.

We then focused on the regulation of DUSP26 phosphatase by AK2. Based on aforementioned data, we hypothesized that AK2 might directly regulate the phosphatase activity of DUSP26. To address this question, DUSP26 protein was purified from bacteria and subjected to *in vitro* phosphatase assays using pNPP. Significant amount of phosphatase activity was readily detected in the purified DUSP26 protein itself (Fig. 3a). Interestingly, the phosphatase activity of DUSP26 protein was enhanced 2- to 3-fold by addition of AK2 protein, depending on the amounts of AK2 protein added. By contrast, addition of AK3 or FADD protein had no effect on the phosphatase activity of DUSP26 (Fig. 3a). AK2 K28E AK-dead mutant protein also enhanced the phosphatase activity of DUSP26 as much as wild-type AK2 did (Fig. 3b). Furthermore, *in vitro* enzyme assays exhibited that the purified DUSP26 protein reduced the amount of p-FADD and this reaction was stimulated by addition of AK2 protein in a synergistic manner (Fig. 3c). These results suggest that DUSP26 functions as a FADD phosphatase and AK2 directly activates the phosphatase activity of DUSP26 independently of its AK activity.

We then characterized the stimulatory effect of AK2 on DUSP26 activity in cells by examining their substrate, p-FADD. Cellular levels of p-FADD, which were significantly reduced by ectopic expression of either DUSP26 or AK2, was severely restrained by co-expression of DUSP26 and AK2 (Fig. 3d). In addition, we found that the reduction of p-FADD by AK2 expression was hampered by the treatment with NSC-87877, a potent inhibitor of DUSP26 (ref. 43)^43^ (Supplementary Fig. 6). What’s more, when we isolated DUSP26 complex with immunoprecipitation assays and compared their enzyme activities, the phosphatase activity of DUSP26 complexes isolated from AK2 knockdown cells was twofold lower than that from control cells (Fig. 3e). Under this condition, DUSP26 complex isolated from control and AK2 knockdown cells contained similar amounts of HA-DUSP26 (Fig. 3e). Furthermore, downregulation of AK2 expression weakened DUSP26-mediated FADD dephosphorylation (Fig. 3f). These observations strongly support the notion that DUSP26 activity is positively regulated by AK2.

### Purified AK2 and DUSP26 form a binary protein complex

**Figure 4:**
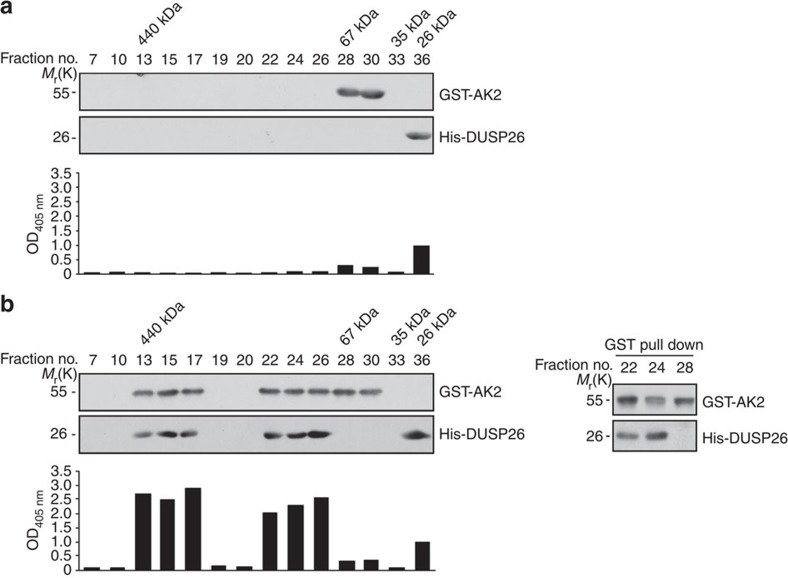
Purified AK2 protein forms a complex with DUSP26 on gel filtration. (**a**) Elusion profile and phosphatase activity of each AK2 or DUSP26 protein on size-exclusion chromatography. Purified GST-AK2 or His-DUSP26 protein (each 10 μg) was separated on a Superdex 200 gel filtration column (0.6-ml fractions). Proteins of each fraction were then separated by SDS–PAGE and analysed by western blotting with anti-AK2 or anti-DUSP26 antibody. Each fraction was also assayed for the phosphatase activity using pNPP. (**b**) The formation of AK2/DUSP26 binary protein complexes on size-exclusion chromatography and stimulation of DUSP26 phosphatase activity by AK2. Purified GST-AK2 and His-DUSP26 proteins (each 10 μg) were combined together for 1 h and then subjected to size-exclusion chromatography as in **a**. Each fraction was examined by western blotting and phosphatase assays. Protein fractions (number 22, 24 and 28) were subjected to GST pull-down assays and the pulled samples were analysed by western blotting with anti-AK2 and anti-DUSP26 antibodies.

To examine the formation of AK2/DUSP26 protein complexes *in vitro*, we carried out a size-exclusion chromatography analysis using Superdex 200. When analysed separately, purified glutatione S-transferase (GST)-AK2 and DUSP26 proteins were detected with their respective molecular weight (His-DUSP26, 26 kDa; and GST-AK2, 56 kDa) (Fig. 4a). Significant phosphatase activity was detected in fraction 36 that contained DUSP26 (Fig. 4a). When incubated together and analysed, however, DUSP26 and AK2 proteins were all detected in two groups of early fractions, fractions 13–17 (~400 kDa) and 22–26 (~100 kDa) (Fig. 4b). Compared with fraction 36 that contained only DUSP26, these two groups showed 2- to 3 times higher phosphatase activity (Fig. 4b). Further, we observed formation of a binary complex containing AK2 and DUSP26 in fractions 22 and 24 (Fig. 4b). These *in vitro* results support our proposal that AK2 forms a protein complex with DUSP26 to enhance the phosphates activity of DUSP26.

### AK2 regulates FADD phosphorylation against cell growth

Based on the previous reports showing that FADD phosphorylation has been associated with cell cycle progression^30^,^32^,^33^, we next evaluated whether AK2 was accountable for FADD-mediated cell cycle alteration. Analysis of HeLa cells at different stages of cell cycle with western blotting revealed that the level of p-FADD was higher at early M phase in AK2 knockdown cells than in control cells (Supplementary Fig. 7a). In support of this, depletion of AK2 in HeLa cells led to significant increase of cell proliferation (Supplementary Fig. 7b). We also assessed the role of DUSP26 in cell cycle alteration and cell growth. Consistently, depletion of DUSP26 in HeLa cells resulted in increase of FADD phosphorylation at early M phase (Supplementary Fig. 7c) and facilitated cell proliferation (Supplementary Fig. 7d). These observations suggest that AK2 and DUSP26 have crucial roles in the regulation of cell proliferation.

**Figure 5:**
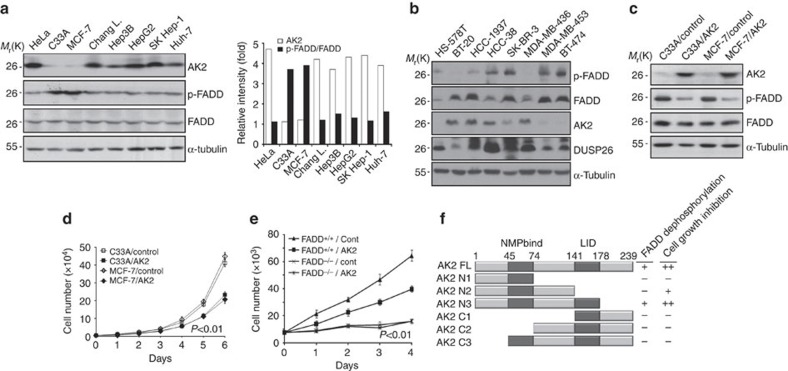
AK2 deficiency is detected in tumour cells with alteration in cell proliferation. (**a**) AK2 expression is downregulated in MCF-7 and C33A tumour cells showing increased p-FADD. The expression profiles of AK2, FADD and p-FADD were examined in cancer cells by western blot analysis. Relative ratios (fold) of AK2 (open box) or p-FADD signal (filled box) to FADD on the blots were determined by densitometric analysis. (**b**) AK2 expression is downregulated in breast tumour cells showing increased level of p-FADD. The expression profiles of indicated proteins were examined in breast cancer cells by western blot analysis. (**c**) Reconstitution of C33A and MCF-7 cells with AK2 reduces FADD phosphorylation. C33A and MCF-7 cells were stably transfected with pcDNA3 (C33A/Control and MCF-7/Control) or AK2 (C33A/AK2 and MCF-7/AK2). Cell extracts were prepared and subjected to western blotting with the indicated antibodies. (**d**) Reconstitution of AK2 results in retardation of cell proliferation in C33A and MCF-7 tumour cells. Cell proliferation of C33A/Control, C33A/AK2, MCF-7/Control, and MCF-7/AK2 stable cells were examined for 6 days. Values are the mean±s.d. (*n*=3). *P*<0.01; *t*-test. (**e**) AK2 suppresses cell proliferation in wild-type MEFs but not in *FADD*^−/−^ MEFs. *FADD*^+/+^ and *FADD*^−/−^ MEFs (7 × 10^3^) were transfected with either pcDNA (Cont) or pAK2-HA and the cell numbers were counted at different time points. Values are the mean±s.d. (*n*=3). *P*<0.01; *t*-test. (**f**) Functional dissection of AK2 domains responsible for FADD dephosphorylation and cell growth inhibition. A schematic diagram of the AK2 deletion mutants and a summary of the FADD dephosphorylation and the cell growth inhibition are described. All of AK2 constructs were fused with GFP.

To address this perspective of AK2 function in detail, we assessed the pathological significance of AK2 function by examining the connection between AK2 dysregulation and cell proliferation in human tumour cells and cancer tissues. Although AK2 expression was detected in most cancer cells, including SK-Hep1 and HepG2 hapatocellular carcinoma cells, its expression was drastically downregulated in MCF-7 breast cancer cells and C33A cervical cancer cells (Fig. 5a). In addition, AK2 expression was also downregulated in other HS-578T, MDA-MB-453, BT-474 and SK-BR-3 breast cancer cells among total eight breast cancer cell lines (Fig. 5b). Interestingly, high levels of p-FADD were detected in MCF-7, MDA-MB-453, BT-474, SK-BR-3 and C33A cells, despite there being no difference in total FADD (Fig. 5a,b). Then, we stably reconstituted MCF-7 and C33A cells with AK2 (MCF-7/AK2 and C33A/AK2 cells, respectively) and examined their FADD phosphorylation status and cell proliferation rates. Compared with MCF-7 and C33A cells, the levels of p-FADD were markedly reduced in MCF-7/AK2 and C33A/AK2 cells (Fig. 5c). Moreover, cell proliferation rates of MCF-7/AK2 and C33A/AK2 cells were significantly suppressed by 40% at 6 day (Fig. 5d). Further, treatment with NSC-87877 exerted a strong inhibitory effect on the proliferation of MCF-7/AK2 cells compared with MCF-7 cells (Supplementary Fig. 8). Collectively, AK2 appears to be a strategic controller that acts to coordinate both cell proliferation and FADD dephosphorylation in these tumour cells.

We next explored whether the cell proliferation–inhibitory activity of AK2 is associated with FADD. When we compared cell proliferation of *FADD*^−/−^ mouse embryo fibroblasts (MEFs) with *FADD*^+/+^ MEFs, *FADD*^*−*/−^ MEFs displayed slower proliferation than *FADD*^+/+^ MEFs (Fig. 5e). In the similar context, when we introduced AK2 into *FADD*^+/+^ and *FADD*^−/−^ MEFs, AK2 restrained cell proliferation of *FADD*^+/+^ MEFs but not that of *FADD*^−/−^ MEFs (Fig. 5e). In addition, ectopic expression of AK2 or AK2 N3 mutant, which retained high activity for FADD dephosphorylation, exhibited growth-suppression activity (Supplementary Fig. 9a). Further, we monitored the effect of p-FADD on cellular proliferation using D4476, a selective inhibitor of CK1 kinase, that is responsible for FADD phosphorylation^42^. Treatment with D4476 reduced the level of p-FADD and suppressed cell proliferation in AK2 knockdown cells (Supplementary Fig. 9b), reversing the stimulatory effect of AK2 knockdown on FADD phosphorylation and cell proliferation. As illustrated in Fig. 5f, it thus appears that AK2 exhibits cell proliferation–inhibitory activity in a FADD-dependent manner.

### AK2 suppresses tumourigenesis and is lost in human cancers

**Figure 6:**
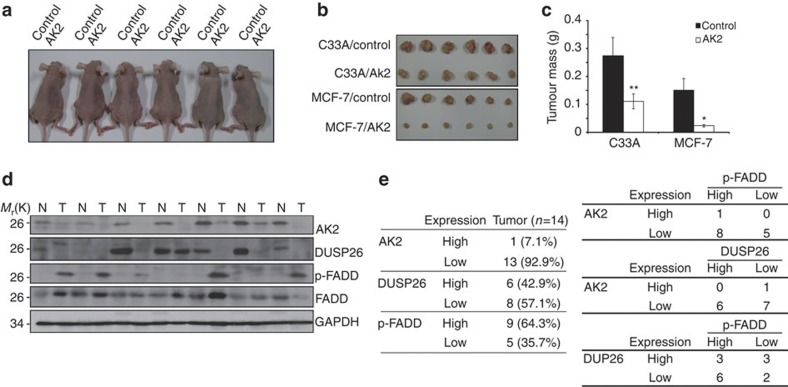
Reduced expression of AK2 increases tumourigenicity *in vivo* and correlates with elevated FADD phosphorylation. (**a**) Reconstitution with AK2 decreases tumourigenicity *in vivo*. C33A/Control and MCF-7/Control cells (1 × 10^6^) were injected subcutaneously into the left side of flank and C33A/AK2 and MCF-7/AK2 cells were injected into the opposite side of the same mice. After tumours had grown to the approved size, tumours were dissected and their sizes (**b**) and volumes were measured (**c**). Values are the mean±s.e.m. (*n*=20). *P<0.05; **P<0.005; *t*-test. (**d**) Loss of AK2 and DUSP26 expression shows the increase of p-FADD in human breast tumours. Tissue extracts were prepared from normal (N) and tumour (T) breast tissues and analysed by western blotting with anti-AK2, anti-p-FADD, anti-FADD and anti-GAPDH antibodies. (**e**) The AK2/DUSP26/p-FADD pathway is manifested in human breast tumours. Summary of the AK2, DUSP26 and p-FADD expression profiles. Inverse correlations between AK2 expression and p-FADD level, as well as DUSP26 expression and p-FADD level in breast specimens.

These observations led us to address whether or not AK2 expression affected tumour growth *in vivo*. After subcutaneous injection of wild-type cells and AK2 over-expression MCF-7 or C33A cells into the flanks of nude mice, we monitored tumour growth for 12 weeks. Compared with control cells, ectopic restoration of AK2 in MCF-7 and C33A cells lacking AK2 expression led to near-complete tumour regression (Fig. 6a–c). Reversely, ablation of endogenous AK2 expression significantly promoted tumour growth in the right side of mouse flank with 70% of substantial tumour progression (Supplementary Fig. 10a). Analysis of the dissected tumours showed that the inoculation of AK2 knockdown cells into nude mice led to a significant increase of tumourigenesis (Supplementary Fig. 10b–d). These results suggest that downregulation of AK2 expression produces impressive *in vivo* tumour efficacy in mice.

In support of this notion, we further extended our analysis to human cancer tissues. Western blotting of 14 human breast cancers showed that AK2 expression was abolished or low in cancer tissues compared with non-cancerous tissues (Fig. 6d). Cancer-specific alleviation of AK2 expression was observed in 13 samples (93%), including cancers in clinical phase I (four cases), II (six cases) and III (three cases) phases, out of the 14 matched tissue sets and DUSP26 expression was lost in 57% of cancers (Fig. 6d,e). On the other hand, p-FADD was apparently elevated in nine samples (64%) out of total 14 cancer tissues, while total FADD was not much changed. Indeed, the levels of p-FADD were high in 62% of cancers showing low levels of AK2 expression and in 75% of cancers showing DUSP26 deficiency (Fig. 6e). Moreover, all of the five cancers (100%) showing high level of p-FADD signal ablated the expression of both AK2 and DUSP26 (Fig. 6d,e), underscoring a strong cross-talk for an inverse correlation between AK2/DUSP26 expression and p-FADD in the human breast cancers.

### AK2^+/−^ MEFs exhibit enhanced p-FADD and cell proliferation

**Figure 7:**
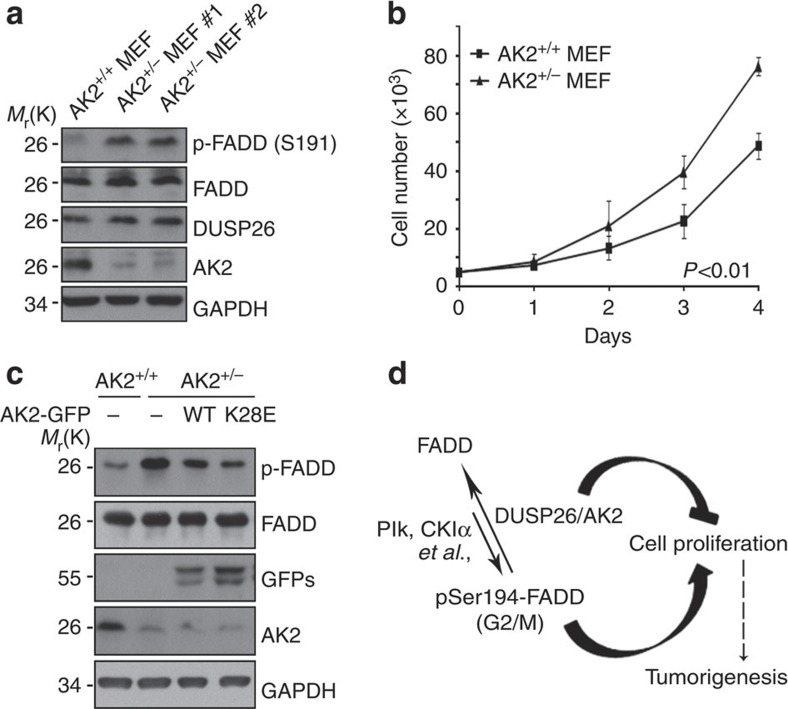
FADD phosphorylation at ser191 and cell proliferation is augmented in *AK2*^+/−^ MEFs. (**a**) Increase of p-FADD (Ser191) in *AK2*^+/−^ MEFs. On embryonic day 9, MEFs were cultured from wild-type and *AK2*^+/−^ mouse embryos. Cell extracts were prepared and subjected to western blotting with anti-mouse pSer_191_-FADD and anti-AK2 antibodies. (**b**) *AK2*^+/−^ MEFs show enhanced cell proliferation. Wild-type and *AK2*^+/−^ MEFs (5 × 10^3^) were prepared and examined every day for cell proliferation for 4 days. Values are the mean±s.d. (*n*=3). *P*<0.01; *t*-test. (**c**) Reconstitution of *AK2*^+/−^ MEFs with AK2 wild-type or AK2 K28E mutant reduces FADD phosphorylation. *AK2*^+/−^ MEF cells were transfected with wild-type AK2-GFP or AK2 K28E-GFP mutant for 24 h and FADD phosphorylation was examined by immunoblotting. (**d**) A proposed model of AK2/DUSP26 protein complex in cell proliferation. AK2 forms a protein complex with DUSP26 to regulate FADD dephosphorylation, and thereby cell proliferation.

To clarify the *in vivo* function of AK2 deficiency in mouse, we generated a line of *AK2* knockout mouse using the gene-trap method^44^. Because homozygous *AK2* knockout (−/−) mouse died *in utero* before embryonic day 7, we prepared MEFs from embryos of heterozygous *AK2* knockout (+/−) mice. By western blot analysis, we confirmed that AK2 expression was reduced in *AK2*^+/−^ MEFs (Fig. 7a). Compared with wild-type MEFs, the phosphorylation of mouse FADD at Ser191 increased in *AK2*^+/−^ MEFs, whereas total FADD and DUSP26 were not changed (Fig. 7a). In addition, the rate of cell proliferation was significantly increased in *AK2*^+/−^ MEFs (Fig. 7b). Consistently, when we expressed AK2 WT or AK2 K28E mutant in *AK2*-null MEFs, FADD phosphorylation was curtailed (Fig. 7c). These results support that as in human tumour cells, AK2 regulates FADD dephosphorylation and cell proliferation in MEFs, showing a conserved role of AK2 in the regulation of cell proliferation and FADD phosphatase activity in mouse tissue.

## Discussion

Functional screening seeking for AK2-associated phosphatase enabled us to identify DUSP26. Our study on it provides several lines of evidence that AK2 is required as an activator for optimal phosphatase activity of DUSP26. First, downregulation of either AK2 or DUSP26 expression enhances FADD phosphorylation. Second, AK2 directly forms protein complex with DUSP26 and enhances the phosphatase activity of DUSP26. Third, AK2 knockdown reduces the phosphatase activity of DUSP26 in cells. Fourth, tumour cells lacking AK2 exhibit high level of p-FADD and reconstitution of AK2 reverses it. Thus, we propose that AK2 is the first positive regulator of DUSP26 phosphatase. AK2/DUSP26 complex described here, however, forms to regulate cell proliferation and is distinct from AK2/FADD/caspase-10 apoptotic complex in that caspase-10 is not present in AK2/DUSP26 complex. Given that DUSP26 functions as a phosphatase of other substrates, including MAP kinases and p53 (refs 14, 15, 17, 19), it is conceivable that the substrate specificity and activity of DUSP26 are variable by and depend on its associated regulator, such as AK2. In addition, the discrepancy of early reports showing distinct roles of DUSP26 in either stimulation^17^ or inhibition^19^,^20^,^21^ of cell growth may also be interpretable, resulting from different expression levels of AK2 in cells analysed.

Other functions of AK2 than AK activity regulate the phosphatase activity of DUSP26. Unlike AK1, the enzymatic activity of AK2 is much low^5^. Then, an important mechanistic question that remains is how AK2 regulates the phosphatase activity of DUSP26. AK2 alone was enough to stimulate DUSP26 activity *in vitro*. It, thus, appears that the binding of AK2 to DUSP26 may induce conformational change of DUSP26 to increase enzyme activity. In addition, our observations that AK2 bound to FADD^5^, as well as DUSP26, and also enhanced the interaction between DUSP26 and FADD imply that AK2 may participate in FADD binding as well to provide DUSP26 with substrate specificity. We believe that FADD is recruited into AK2/DUSP26 complex as a substrate. Consistent with that notion, other proteins, such as heat-shock transcription factor 4b and a motor subunit of the KIF3, also bind to DUSP26 as substrates^15^,^18^. It will thus be interesting to examine whether AK2 is also required for DUSP26 to regulate these proteins.

It appears that FADD phosphorylation is irrelevant to cell death. Instead, FADD very much represented the role of AK2 in the regulation of cell proliferation. This multi-functionality of FADD may depend primarily on its subcellular location^31^. FADD shuttles between the cytosol and the nucleus^32^,^45^ and this signal is unclear; however, FADD trafficking requires phosphorylation of the protein on Ser194 (ref. 46). As predicted, AK2 knockdown increased p-FADD detected in the nucleus, illustrating an opposite role of AK2 in FADD trafficking. Although AK2 is mainly located in the mitochondria, substantial amount of AK2 is also found in the nucleus. We identified at least two nuclear localization signals located in its middle (residues 74–141) and C-terminal (residues 141–239) regions of AK2. Paradoxically, mitochondria-targeted AK2 did not affect FADD phosphorylation. Therefore, it appears that AK2 present in the non-mitochondria, such as nucleus, regulates cell proliferation. As reported^13^, we also detected DUSP26 in the nucleus as well as in the cytosol.

In human cells arrested in G1/S phase, FADD is unphosphorylated, but it is phosphorylated exclusively at Ser194 in cells arrested at G2/M phase^34^,^47^. Despite some controversial results, FADD dephosphorylation may be required for the exit from M phase. Thus, ectopic expression of FADD S194D phospho-mimic mutant results in abnormal accumulation of cells in G2/M phase^47^. Consistent to that finding, the binding of AK2 to DUSP26 was enhanced in nocodazole-treated and G2/M phase-arrested cells, providing a hint on the mechanism that regulates DUSP26 activity during cell cycle progression.

As a crucial regulator of DUSP26, AK2 has an essential role in cell proliferation and tumourigenesis. Thus, downregulation of AK2 expression seen here in the human breast cancers may regulate cell proliferation that is contributed by the phosphorylation of their substrate, FADD. Dysregulation of this AK2/DUSP26/FADD signalling pathway does not have to be limited to breast cancer. We also observed significant downregulation of AK2 in other cancers, such as liver and lung cancers. We, therefore, propose that AK2 is a negative regulator of tumour growth that activates DUSP26 to suppress cell proliferation by FADD in human cancers (Fig. 7d).

## Methods

### Cell culture and antibodies

HeLa, HEK293T, C33A and MCF-7 cells (The American Type Culture Collection (ATCC), Manassas, VA, USA) were cultured in Dulbecco’s Modified Eagles Medium (DMEM; HyClone, South Logan, UT, USA) supplemented with 10% fetal bovine serum (HyClone). Wild-type and *FADD* knockout MEFs were cultured with DMEM with 10% fetal bovine serum. Polyclonal rabbit anti-AK2 (Santa Cruz Biotechnology, CA, USA; sc-28786) antibody was described previously^5^. Monoclonal mouse anti-FADD (BD Biosciences, San Jose, CA, USA; BD556402), anti-phospho-FADD (Cell Signaling, Beverly, MA, USA; 2781), anti-DUSP26 (Genetax; GTX109283), anti-GFP (Santa Cruz Biotechnology; sc-8334), anti-PARP (Santa Cruz Biotechnology; sc-8007), anti-α-tubulin (Sigma-Aldrich, St Louis, MO, USA), and anti-Flag (Sigma-Aldrich; F1804) antibodies were used in western blot analysis (1 μg ml^−1^). Transfection was carried out with LipofectAMINE reagent (Invitrogen, Carlsbad, CA, USA) following the manufacturer’s instructions. For generation of stable cell lines, HeLa cells were transfected with vector using LipofectAMINE reagent (Invitrogen) for 24 h and then grown in selection medium containing 1 mg ml^−1^ G418 (Invitrogen) for 2 weeks. C33A and MCF-7 cells were also transfected with vector and selected for 2 weeks in the presence of 1.2 mg ml^−1^ G418. After single cell cloning, the clones were screened by western blot analysis. HEK293T cells were used for the screening and immunoprecipitation assays for maximum efficiency of DNA transfection and the other tumour cells were analysed for cell proliferation.

### Plasmid constructs

Construction of pAK2 shRNAs was described previously^5^. For construction of pDUSP26 shRNA, the forward and reverse 60-nucleotide fragments containing sequences of human DUSP26 (shRNA no. 1–5′, 5′-gatccccgagaccaggacatggctaattcaagagattagccatgtcctggtctcttttta-3′; shRNA no. 1–3′, 5′-agcttaaaaagagaccaggacatggctaatctcttgaattagccatgtcctggtctcggg-3′; shRNA no. 2–5′, 5′-gatccccccatcaagaaagtcaaagattcaagagatctttgactttcttgatggttttta-3′; shRNA no. 2–3′, 5′-agcttaaaaaccatcaagaaagtaaaagatctcttgaatctttgactttcttgatggggg-3′) were synthesized, annealed and cloned into the *Bgl*II and *Hin*dIII sites of pSuper mammalian expression vector (OligoEgine, Seattle, WA, USA). The cDNAs encoding AK1, AK2 and AK3 were obtained by PCR and cloned into pcDNA3-HA. The cDNAs encoding AK2 FL, AK2 N1, AK2 N2, AK2 N3, AK2 C1, AK2 C2 and AK2 C3 were cloned into the *Xho*I site of pEGFP (enhanced green fluorescent protein) N1. DUSP26 cDNA was also obtained by PCR and cloned into the *Eco*RI and *Eco*RV sites of p3 × FLAG-CMV-10 vector (Sigma-Aldrich).

### Identification of DUSP26-binding proteins by LC-MS/MS

DUSP26-binding proteins were affinity-purified from extracts of HEK293T cells stably expressing 3 × Flag-tagged DUSP26. The DUSP26-binding proteins were immunoprecipitated using anti-Flag antibody-conjugated agarose beads (Sigma-Aldrich) from extracts that were washed with buffer containing 20 mM Tris–HCl, pH 7.9, 15% glycerol, 1 mM EDTA, 1 mM dithiothreitol (DTT), 0.2 mM phenylmethylsulfonyl fluoride (PMSF), 0.05% Nonidet P40 and 150 mM KCl to remove non-specific contaminants. The bound proteins were eluted by competition with the Flag peptide (0.1 mg ml^−1^), resolved by SDS–PAGE and prepared for LC-MS/MS analysis. Peptide samples were injected into a column by a Surveyor autosampler (Surveyor; Thermo Finnigan, San Jose, CA, USA) and separated by C18 column. The eluent was directly transferred to the electrospray ionization source of a Thermo Finnigan LCQ DecaXPplus ion trap mass spectrometer. Automated peak recognition, dynamic exclusion, and daughter ion scanning of the two most intense ions were performed and analysed by the XCALIBUR software.

### *In vitro* FADD phosphatase assay

His-FADD was purified from bacteria and incubated with Plk in a kinase reaction buffer (20 mM Tris-HCl, pH 7.5, 10 mM MgCl_2_, 0.01% Triton X-100, 0.5 mM Na_3_VO_4_, 2.5 mM DTT and 200 mM ATP). The reaction products were then incubated with purified GST-AK2 or His-DUSP26 (5 μg) in the phosphatase reaction buffer (50 mM Tris–HCl, pH 7.4, 150 mM NaCl, 5 mM MgCl_2_ and 0.02% Triton X-100). After 1 h at 37 °C, the reaction products were separated by SDS–PAGE and analysed by western blot analysis.

### pNPP hydrolysis assay

Samples were incubated with pNPP (Sigma-Aldrich) in reaction buffer (25 mM HEPES, pH 7.2, 50 mM DTT and 2.5 mM EDTA) for 30 min at 25 °C and then centrifuged briefly. The pNPP hydrolysis was assessed by measuring the OD_405 nm_ of the reaction supernatants with spectrometer.

### Cytosolic–nuclear fractionation assay

For separation of the cytosolic or nuclear fraction, HeLa and HEK293T cells were harvested, resuspended in buffer A (25 mM Tris–HCl, pH 8.0, 10 mM KCl, 1 mM DTT and 0.5 mM PMSF) and incubated for 15 min on ice. After cell lysis elicited by the addition of 0.5% NP-40 and vigorous vortexing, the lysates were spun down at 1,400 *g* for 1 min, and the supernatants were saved as the cytosolic fractions. Nuclear fractions were obtained by resuspending and vortexing the pellets in buffer C (50 mM Tris–HCl, pH 8.0, 400 mM NaCl, 1 mM DTT and 1 mM PMSF) followed by incubation for 30 min on ice. After centrifugation at 15,000 *g* for 30 min at 4 °C, the supernatants were collected as the nuclear fractions.

### RT–PCR

Total cellular RNA was isolated from cells using Trizol reagent (MRC, Cincinnati, OH, USA). The following primer sets for DUSP26 were then used for PCR (25 cycles): 5′-atgtgccctggtaactgg-3′ and 5′-tcgccaccggctgtgtga-3′ and for GAPDH: 5′-gaaggtgaaggtcggagtca-3′ and 5′-gttcacacccatgacgaaca-3′.

### Chromatographic methods

HeLa cells were lysed by homogenization in hypertonic buffer (50 mM PIPES, 2 mM EDTA, 5 mM DTT, 15 mM MgCl_2_, 50 mM KCl, 0.1% CHAPS and protease inhibitors). Cell extracts were then fractionated by size-exclusion chromatography on Superdex 200 analytical columns using a fast protein liquid chromatography protein purification system (Amersham Pharmacia Biotech, Herts, UK). Superdex columns were eluted at 4 °C with buffer (pH 7.0) containing 5% (w/v) sucrose, 0.1% (w/v) CHAPS, 20 mM HEPES/NaOH and 5 mM DTT. The columns were calibrated with protein standards (Amersham Pharmacia Biotech).

### Generation of *AK2* knockout mice

*AK2* gene-trapped embryonic stem cells (RRR133) were provided by BayGenomics of the International Gene Trap Consortium. AK2 heterozygous mouse was generated by following the protocol provided by BayGenomics. Genotypes were analysed using genomic PCR with synthetic oligonucleotides for β-galactosidase (β-gal-5′, 5′-ttattatcatcgatgagcgtggtggtggttatgc-3′; β-gal-3′, 5′-gcgcgtacatcgggcaaataatatc-3′) and for AK2 (AK2-5′, 5′-ttatcgatgagcgtggtggttatgc-3′ and AK2-3′, 5′-gcgcgtacatagggcaaataatatc-3′).

### Xenograft assay

The flank and rump of the four-week-old male nude mice were injected subcutaneously with tumour cells (1 × 10^6^). Tumour growth was monitored every week. All experiments involving animals were performed according to protocols approved by the Seoul National University Institutional Animal Care and Use Committee guidelines.

### Human cancer tissue analysis

The biospecimens for this study were provided by the Biobank of Chonbuk National University Hospital, a member of the National Biobank of Korea, which is supported by the Ministry of Health, Welfare and Family Affairs. All samples derived from the National Biobank of Korea were obtained with informed consent under institutional review board-approved protocols.

### Cell growth assay

To measure cell density using DNA content, 3 × 10^3^ cells were seeded into 96-well plates, after which cells were removed every day and immediately frozen at −70 °C. Cells were then thawed and lysed, and DNA content was measured using a CyQUANT Cell Proliferation Assay Kit (Invitrogen) by measuring the fluorescent signal (excitation, 480 nm; and emission, 520 nm).

### *In vitro*-binding assay

Purified GST-fusion proteins coupled to Glutathione-Sepharose 4B (Amersham Biosciences) were incubated for 3 h in binding buffer [50 mM Tris–HCl (pH 6.8), 100 mM NaCl, 1 mM DTT, 2 mM EDTA, 0.05% Triton X-100, 1 mM PMSF, and 5% (v/v) glycerol] at 4 °C with [^35^S]-methionine-labeled proteins which were *in vitro* translated using a TNT-coupled transcription/translation system (Promega, Madison, WI, USA). Typically, 20 μg of the immobilized fusion protein was incubated with 10 μl of *in vitro* translated sample. After pull-down assay with Glutathione-Sepharose beads, the precipitated proteins were extensively washed with binding buffer, separated by 12% SDS–PAGE, and detected by autoradiography.

### Two-dimensional gel electrophoresis

Cells were washed three times with ice-cold PBS, after which total cell lysates were prepared by sonication in lysis buffer (9 M urea, 2% CHAPS, 0.8% pharmalyte and 1% DTT). The lysates were centrifuged at 15,000 *g* for 30 min, after which the supernatant was collected and stored at −80 °C. Samples were applied to 18-cm long pH 4–7 IPG strips (GE Healthcare, Piscataway, NJ) for 12 h at 10 V using IPGphor (GE Healthcare). Isoelectric focusing was performed for 1 h at 500 V and for 1 h at 1,000 V, after which the voltage was gradually increased to 8,000 V for 1 h. After focusing at 8,000 V for an additional 4 h, each strip was incubated for 20 min in buffer containing 50 mM Tris-HCl (pH 8.8), 6 M urea, 30% glycerol, 2% SDS and 1% DTT, and then for an additional 20 min in the same buffer containing 2.5% indoleacetic acid (IAA) instead of DTT. The equilibrated strip was placed onto 10–15% gradient polyacrylamide gel and the gel was electrophoresed at 70 V in running buffer (25 mM Tris-HCl, pH 8.8, 192 mM glycine and 0.1% SDS).

### *In vitro* phosphatase assay using HPLC

Synthetic FADD (GAMSPMS) and p-FADD peptides (GAMpSPMS) (each 20 μg) (Peptron Inc., Daejeon, Korea) were left untreated or incubated with purified AK2 protein (8 μg) for 30 min at 37 °C. Then, the reaction products were analysed by HPLC reverse phase.

### FACS analysis

HeLa cells were spun down, washed with PBS and fixed in 70% ethanol. After incubation for 10 min on ice, cells were centrifuged at 800*g* for 10 min at 4°C and then washed three times with ice-cold PBS. The pellets were resuspended in 500 μl of PBS and stained with 50 μg ml^−1^ propidium iodide (Sigma-Aldrich) in the presence of 100 μg ml^−1^ RNase (Invitrogen). Samples were then analysed using a FACSCalibur flow cytometer (BD Biosciences).

## Author contributions

H.-J.K., H.-J.L. and Y.-K.J. conceived and designed the experiments. H.-J.K., H.-J.L., Y.-M.O., S.-G.C., S.-H.H., H.-J.K., S.-Y.L. and J.-W.C. performed the experiments. H.-J.K., H.-J.L., K.-S.K. and Y.-K.J. analysed the data. D.S.H., K.-S.K., H.-J.K., J.Z., H.-J.Y. and D.-Y.N. contributed reagents/materials/analysis tools. H.-J.K., H.-J.L. and Y.-K.J. wrote the paper. Y.-K.J. conceived and designed the study.

## Additional information

**How to cite this article:** Kim, H. *et al.* The DUSP26 phosphatase activator adenylate kinase 2 regulates FADD phosphorylation and cell growth. *Nat. Commun.* 5:3351 doi: 10.1038/ncomms4351 (2014).

## Supplementary Material

Supplementary Information Supplementary Figures 1-10
