# Supplementary material for: The DUSP26 phosphatase activator adenylate kinase 2 regulates FADD phosphorylation and cell growth
Source: Nat Commun. 2014 Feb 19;5:3351. doi: 10.1038/ncomms4351 (PMC3948464; doi:10.1038/ncomms4351)
Supplement: Supplementary Information — Supplementary Figures 1-10 [file ncomms4351-s1.pdf]

Supplementary Information

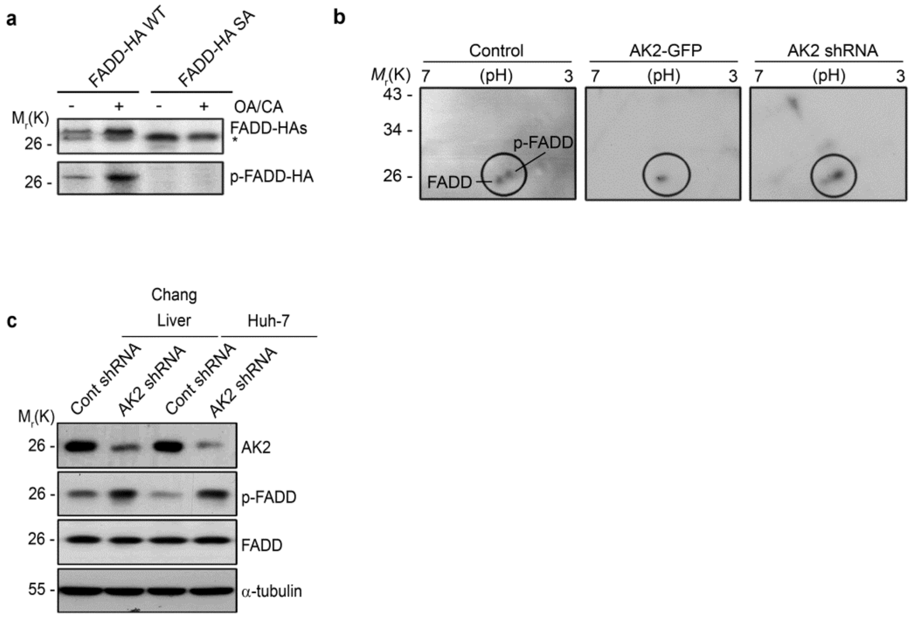

Supplementary Figure 1. AK2 induces FADD dephosphorylation. (a) Detection of FADD phosphorylation at Ser 194. HeLa cells were transfected with pFADD-HA or pFADD S194A-HA for 24 h, after which they were left untreated or incubated with okadaic acid (OA, 5 μM) and calyculin A (CA, 50 nM) for 2 h. Cell extracts were prepared and examined by western blotting with anti-HA and anti-pSer$_{194}$-FADD antibodies. The asterisk (*) indicates faster migrating FADD. **(b)** HeLa cells were transfected with pGFP (Control), pAK2-GFP or pAK2 shRNA for 24 h and cell extracts were subjected to 2D-PAGE and western blotting with anti-FADD antibody. **(c)** AK2 regulates FADD phosphorylation in Chang liver and Huh-7 cells. Chang liver and Huh-7 cells were transfected with pAK2 shRNA for 36 h, after which cell extracts were subjected to western blotting with the indicated antibodies.

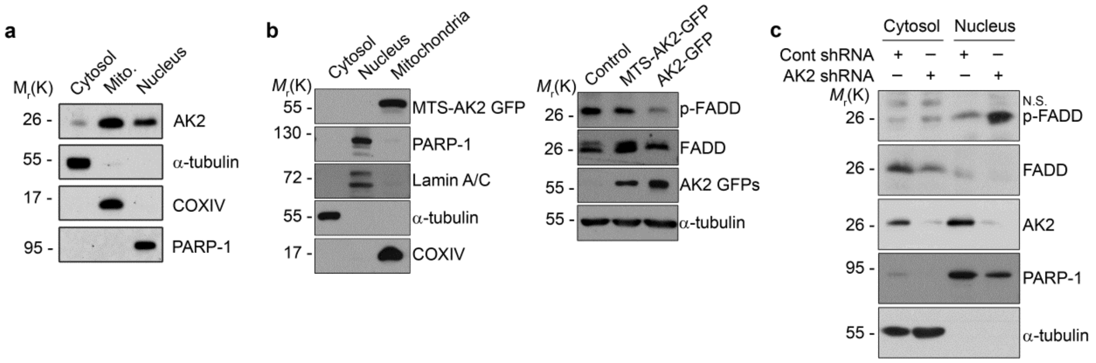
a
Mr(K)
Cytosol
Mito.
Nucleus
26 -
55 -
17 -
95 -
AK2
α-tubulin
COXIV
PARP-1
b
Cytosol
Nucleus
Mitochondria
Mr(K)
55 -
130 -
72 -
55 -
17 -
MTS-AK2 GFP
PARP-1
Lamin A/C
α-tubulin
COXIV
Control
MTS-AK2-GFP
AK2-GFP
26 -
26 -
55 -
55 -
p-FADD
FADD
AK2 GFPs
α-tubulin
c
Cytosol
Nucleus
Cont shRNA
AK2 shRNA
Mr(K)
N.S.
26 -
26 -
26 -
95 -
55 -
p-FADD
FADD
AK2
PARP-1
α-tubulin

Supplementary Figure 2. Nuclear AK2, but not mitochondrial AK2, regulates FADD phosphorylation. (a) HeLa cell extracts were subjected to fractionation analysis by centrifugation, after which the cytosolic (Cytosol), mitochondrial (Mito.), and nuclear (Nucleus) fractions were collected and analyzed by western blotting with the indicated antibodies. **(b)** Mitochondria-targeted AK2 fails to reduce p-FADD level in cells. Mitochondria targeting sequence (MTS) is from subunit VIII of human cytochrome *c* oxidase[5]. HeLa cells were transfected with pAK2-GFP or pMTS-AK2-GFP for 24 h and cell extracts were subjected to western blotting using the indicated antibodies or fractionated by centrifugation into cytosol, nucleus, and mitochondria. The fractions were proved by western blotting with the indicated antibodies. PARP-1, α-tubulin, and COXIV were served as markers for the nucleus, cytosol, and mitochondria, respectively. **(c)** FADD phosphorylation is regulated by AK2 in the nucleus. HeLa/Cont shRNA and HeLa/AK2 shRNA cell extracts were separated into cytosolic and nuclear fractions. The fractions were subjected to western blot analysis with the indicated antibodies.

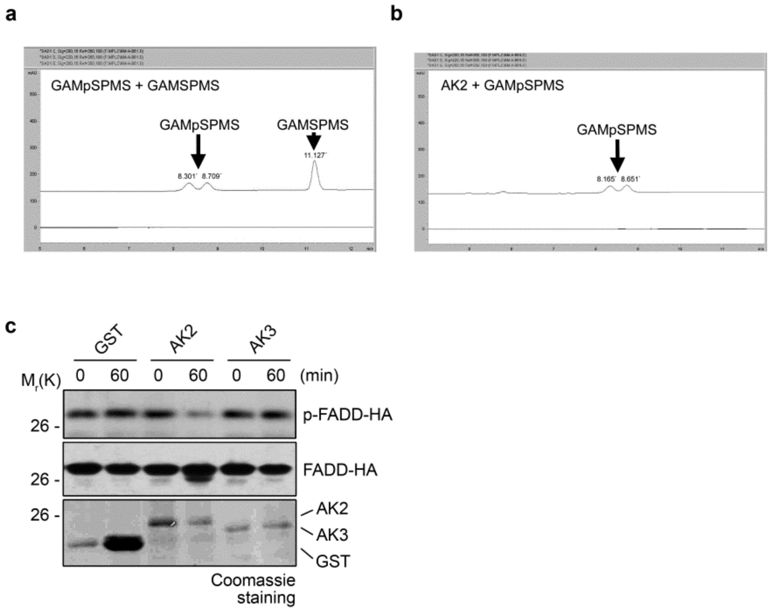
a
GAMpSPMS + GAMSPMS
GAMpSPMS
GAMSPMS
8.301
8.709
11.127
b
AK2 + GAMpSPMS
GAMpSPMS
8.165
8.651
c
GST
AK2
AK3
M_r(K)
0
60
0
60
0
60
(min)
26 -
p-FADD-HA
26 -
FADD-HA
26 -
AK2
AK3
GST
Coomassie staining

Supplementary Figure 3. Purified AK2 protein exhibits no phosphatase activity *in vitro* but stimulates FADD dephosphorylation in the FADD-containing cellular complex. (a,b) Lack of phosphatase activity in purified AK2 protein. A synthetic FADD peptide (GAMSPMS) and a p-FADD peptide (GAMpSPMS) were left untreated (a) or incubated with bacterially purified AK2 protein (8 μg) (b) and then subjected to HPLC reverse phase analysis. **(c)** Purified AK2 protein enhances the FADD dephosphorylation of the FADD-containing complexes. HEK293T cells were transfected with pcDNA-HA or pFADD-HA for 24 h, after which cell extracts were subjected to immunoprecipitation (IP) assay using anti-HA antibody. The immunocomplex was incubated with purified GST, His-AK2 or His-AK3 protein (each 10 μg) for 1 h. Then, the reactions were analyzed by western blotting with anti-p-FADD and anti-FADD antibodies. The purified proteins used in these assays were stained with coomassie-blue.

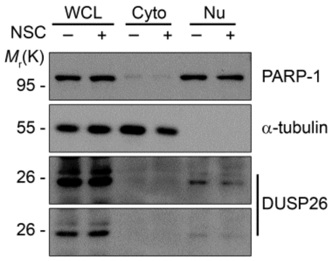
WCL
Cyto
Nu
NSC
–
+
–
+
–
+
M_r(K)
95 -
55 -
26 -
26 -
PARP-1
α-tubulin
DUSP26

Supplementary Figure 4. Localization of DUSP26 in the nuclear fraction. Cell extracts were treated with NSC (50 μM), an inhibitor of DUSP26, for 1 h and then separated into cytosolic and nuclear fractions as described in Supplementary Figure 2c. The fractions were collected and analyzed by western blotting with the indicated antibodies.

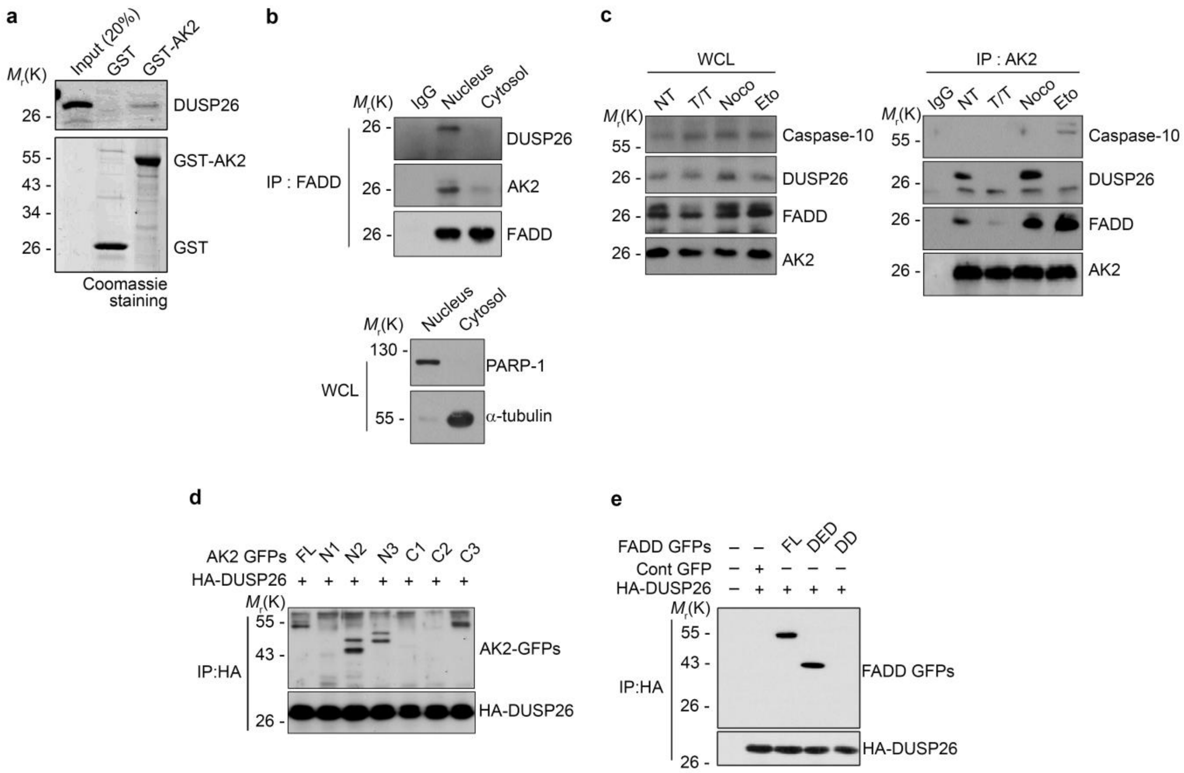
a
Input (20%)
GST
GST-AK2
Mr(K)
26 -
DUSP26
55 -
43 -
34 -
26 -
GST-AK2
GST
Coomassie staining
b
Mr(K)
IgG
Nucleus
Cytosol
IP : FADD
DUSP26
AK2
FADD
WCL
PARP-1
α-tubulin
130 -
55 -
c
WCL
NT
T/T
Noco
Eto
Caspase-10
DUSP26
FADD
AK2
IP : AK2
IgG
d
AK2 GFPs
FL
N1
N2
N3
C1
C2
C3
HA-DUSP26
IP:HA
AK2-GFPs
HA-DUSP26
e
FADD GFPs
Cont GFP
HA-DUSP26
DED
DD
FADD GFPs

Supplementary Figure 5. AK2 binds to DUSP26 *in vitro* and in cells. (a) *In vitro* binding of AK2 to DUSP26. Purified GST-AK2 protein was incubated with *in vitro*-translated DUSP26 in the presence of [^{35}S]-methionine. The input and retained proteins were then separated by SDS-PAGE and visualized by autoradiography or coomassie-blue staining. **(b)** Formation of endogenous AK2/FADD/DUSP26 protein complexes in the nucleus. HEK293T cell extracts were subjected to fractionation analysis, after which the nuclear and cytosolic fractions were collected and subjected to immunoprecipitation (IP) assay using anti-FADD antibody. The immunoprecipitates were analyzed by western blotting with anti-FADD, anti-AK2, and anti-DUSP26 antibodies. Nuclear and cytoplasmic fractions were confirmed by western blotting with anti-PARP-1 and anti-α-tubulin antibodies. **(c)** Increment of AK2 and DUSP26-containing protein complex in cells exposed to nocodazole. HeLa cells were left untreated or treated with double thymidine (2 mM) for 36 h, nocodazole (50 ng/ml) for 6 h, or etoposide (100 μM) for 12 h. Cell extracts prepared were subjected to immunoprecipitation (IP) assay using anti-AK2 antibody. **(d)** The middle region of AK2 containing NMPbind is required for its binding to DUSP26. HEK293T cells were co-transfected with HA-DUSP26 and various AK2-GFP deletion mutants for 24 h, after which cell extracts were subjected to immunoprecipitation (IP) using anti-HA antibody. **(e)** DUSP26 binds to death effector domain of FADD. HEK293T cells were co-transfected with pHA-DUSP26 and either GFP, GFP-FADD or GFP-FADD deletion mutant for 24 h. Cell extracts were subjected to immunoprecipitation (IP) assay with anti-HA antibody. The immunoprecipitates were examined by western blotting with anti-HA and anti-GFP antibodies.

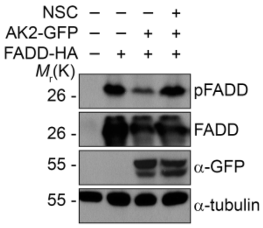
NSC – – – +
AK2-GFP – – + +
FADD-HA – + + +
M_r(K)
26 -
pFADD
26 -
FADD
55 -
α-GFP
55 -
α-tubulin

Supplementary Figure 6. Requirement of DUSP26 activity in AK2-mediated FADD dephosphorylation. HEK293T cells were transfected with AK2-GFP and/or FADD for 24 h and then left untreated or exposed to 10 μM NSC-87877 for 6 h. Cells were then analyzed with immunoblotting using the indicated antibodies.

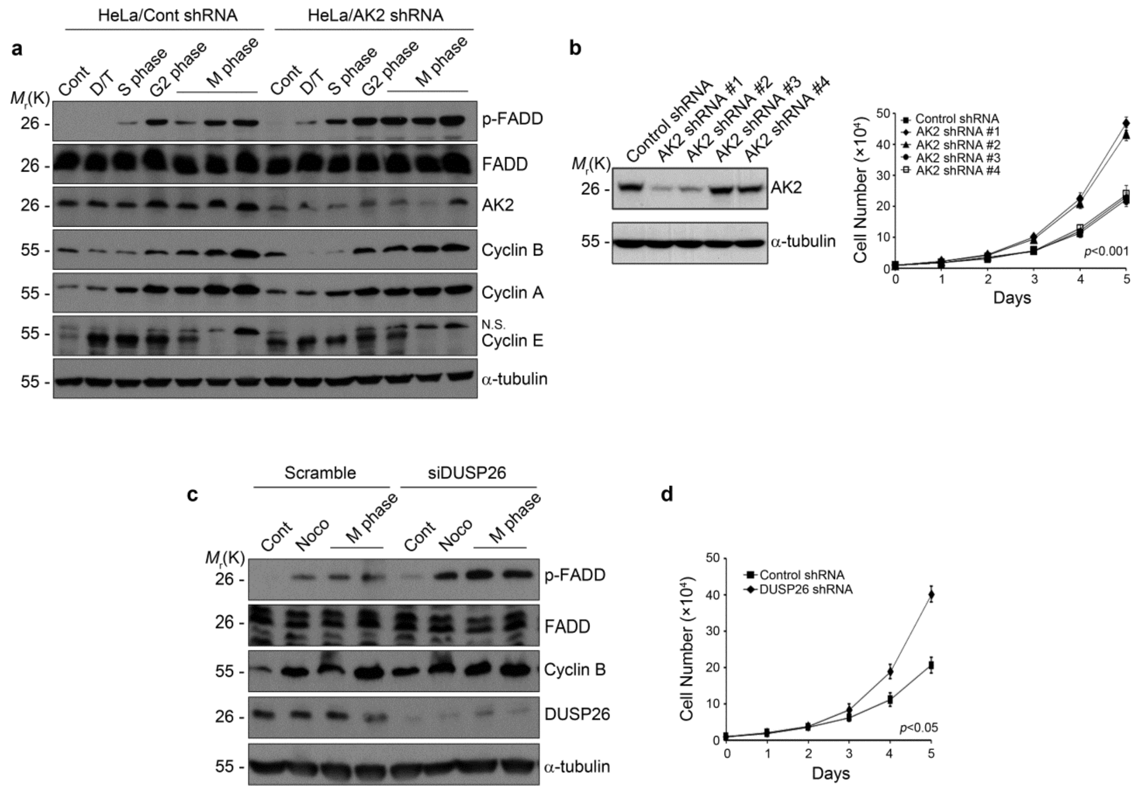
a
HeLa/Cont shRNA
HeLa/AK2 shRNA
Cont
D/T
S phase
G2 phase
M phase
M_r(K)
26 -
55 -
p-FADD
FADD
AK2
Cyclin B
Cyclin A
N.S.
Cyclin E
α-tubulin
b
Control shRNA
AK2 shRNA #1
AK2 shRNA #2
AK2 shRNA #3
AK2 shRNA #4
AK2
α-tubulin
Cell Number (×10⁴)
Days
p<0.001
c
Scramble
siDUSP26
Cont
Noco
M phase
p-FADD
FADD
Cyclin B
DUSP26
α-tubulin
d
Control shRNA
DUSP26 shRNA
Cell Number (×10⁴)
Days
p<0.05

Supplementary Figure 7. AK2 and DUSP26 regulate cell proliferation. (a) Reduction of AK2 expression increases p-FADD in G2/M phase. HeLa/Cont shRNA and HeLa/AK2 shRNA cells were treated with 2 mM double thymidine block for 36 h. At various time points after release from the block, cells were harvested and cell extracts were subjected to western blotting using the indicated antibodies. **(b)** Down-regulation of AK2 expression enhances cell proliferation. HeLa/Cont shRNA and HeLa/AK2 shRNA cells (1×10^4) per well in 6-well plates were prepared and cultured for 5 days. The number of cells was then counted at the indicated days. The expression profile of AK2 in cells was examined by western blot analysis. Values are the mean ± S.D. ($n = 3$). $P < 0.001$; *t*-test. **(c)** Elevation of p-FADD level by DUSP26 knockdown. HeLa cells were transfected with scrambled siRNA or DUSP26 siRNA for 36 h and treated with 50 ng/ml nocodazole for 12 h. Cells were then released from the cell cycle block and cell extracts prepared at the indicated phase of cell cycle were analyzed with western blotting. **(d)** Down-regulation of DUSP26 expression increases cell proliferation. HeLa cells (1×10^3) were transfected with pSuper or pDUSP26 shRNA for 36 h and then cultured for 5 days. The numbers of cells were counted at the indicated days. Values are the mean ± S.D. ($n = 3$). $P < 0.05$; *t*-test.

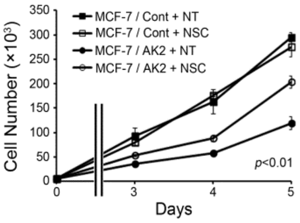
MCF-7 / Cont + NT
MCF-7 / Cont + NSC
MCF-7 / AK2 + NT
MCF-7 / AK2 + NSC
Cell Number (×10³)
350
300
250
200
150
100
50
0
0
3
4
5
Days
p<0.01

Supplementary Figure 8. Requirement of DUSP26 in the AK2-mediated regulation of cell proliferation. Stable MCF-7/control or MCF-7/AK2 cells were left untreated (NT) or treated with 10 μM NSC-8787 for 6 h and then cell proliferation was measured for the indicated times. Values indicate the mean values ± S.D. (*n* = 3). $P < 0.01$; *t*-test.

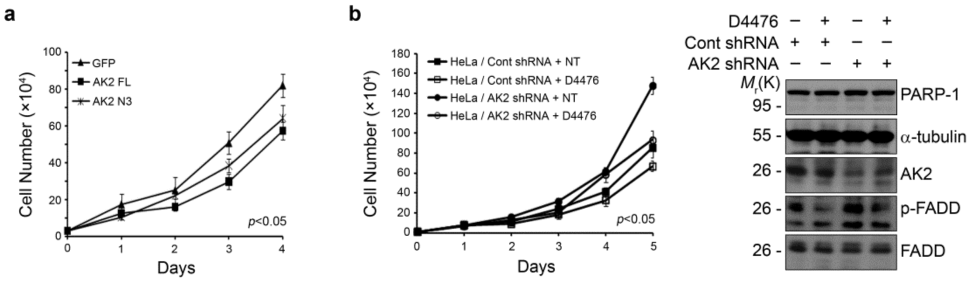
a
b
GFP
AK2 FL
AK2 N3
Cell Number (×10⁴)
Days
p<0.05
HeLa / Cont shRNA + NT
HeLa / Cont shRNA + D4476
HeLa / AK2 shRNA + NT
HeLa / AK2 shRNA + D4476
D4476
Cont shRNA
AK2 shRNA
Mr(K)
PARP-1
α-tubulin
AK2
p-FADD
FADD

Supplementary Figure 9. Contribution of AK2 and DUSP26 to cell proliferation. (a) Capacity of AK2 deletion mutants to regulate cell proliferation. HeLa cells were transiently transfected for 24 h with the indicated AK2 constructs and then subjected to cell proliferation assays. Values are the mean ± S.D. (n = 3). $P < 0.05$; *t*-test. **(b)** AK2 regulates the cell proliferation via FADD phosphorylation. After pretreatment with or without CK inhibitor D4476 (50 μM) for 6 h, HeLa/Cont shRNA and HeLa/AK2 shRNA cells were subjected to cell proliferation assay and western blotting using the indicated antibodies (n = 3). $P < 0.05$; *t*-test.

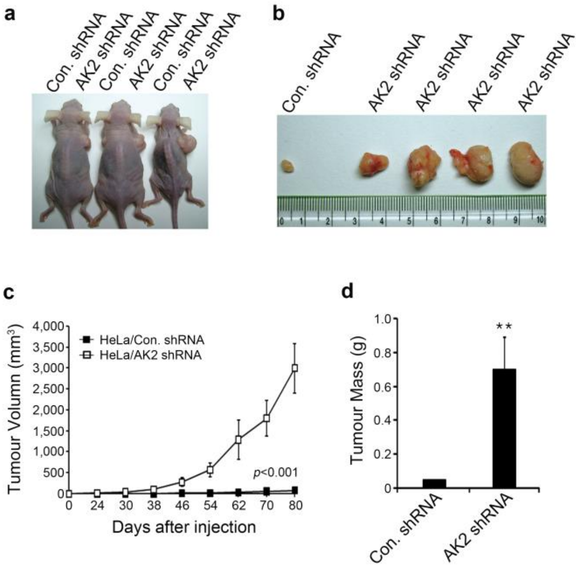
a
Con. shRNA
AK2 shRNA
Con. shRNA
AK2 shRNA
Con. shRNA
AK2 shRNA
b
Con. shRNA
AK2 shRNA
AK2 shRNA
AK2 shRNA
AK2 shRNA
c
Tumour Volumn (mm3)
HeLa/Con. shRNA
HeLa/AK2 shRNA
p<0.001
Days after injection
d
Tumour Mass (g)
**
Con. shRNA
AK2 shRNA

Supplementary Figure 10. Reduced expression of AK2 increases tumorigenicity *in vivo* and is found in cancer tissues showing elevated FADD phosphorylation. (a-d) Xenograft assay showing the increased growth of AK2 knockdown cells *in vivo.* Four-week-old male Nude mice were used to examine the tumorigenicity of AK2 knockdown cells. In xenograft assay, HeLa/Cont shRNA cells (1×10^6) were injected subcutaneously into the left side of flank (7 mice) or rump (3 mice) of 10 mice and HeLa/AK2 shRNA cells were injected into the opposite side of the same mice (a). Tumor growth was monitored every week for 12 weeks and measured by using a ruler (b). After tumors had grown to the approved size, mice were sacrificed. Tumors were dissected and their sizes (c) and volumes were measured (d). Values are the mean ± S.E.M. (n=10). $^{**}P < 0.005$; *t*-test.
